# Do Tumour Size, Type and Localisation Affect Resection Rate in Patients with Spinal Schwannoma?

**DOI:** 10.3390/medicina58030357

**Published:** 2022-03-01

**Authors:** Ahmet Parlak, Marvin Darkwah Oppong, Ramazan Jabbarli, Oliver Gembruch, Philipp Dammann, Karsten Wrede, Laurèl Rauschenbach, Ulrich Sure, Neriman Özkan

**Affiliations:** Department of Neurosurgery and Spine Surgery, University Hospital Essen, 45122 Essen, Germany; marvin.darkwahoppong@uk-essen.de (M.D.O.); ramazan.jabbarli@uk-essen.de (R.J.); oliver.gembruch@uk-essen.de (O.G.); philipp.dammann@uk-essen.de (P.D.); karsten.wrede@uk-essen.de (K.W.); laurel.rauschenbach@uk-essen.de (L.R.); ulrich.sure@uk-essen.de (U.S.); neriman.oezkan@uk-essen.de (N.Ö.)

**Keywords:** spinal schwannoma, peripheral nerve sheath tumours, gross total resection, recurrence rate, subtotal resection

## Abstract

*Background and Objectives:* Spinal schwannomas are benign tumours that can present with various symptoms such as pain, radiculopathy and neurological deficit. Gross total resection (GTR) is of key importance for local recurrence. The aim of this study is to describe the clinical characteristics, resection rate, clinical outcome, as well as tumour recurrence, in patients with non-syndromic spinal schwannomas and to clarify which factors affect the resection rate. *Materials and Methods:* Patients with non-syndromic spinal schwannomas that underwent surgical resection between January 2009 and December 2018 at a single institution were included. Demographic parameters, clinical symptoms, tumour localisation and size, surgical approach and complications were noted. Factors influencing the extent of resection, the surgeon’s decision regarding the approach and the occurrence of new postoperative deficits were evaluated. *Results:* Fifty patients (18 females) were included. The most common presenting symptom was radiculopathy (88%). The lumbar spine was the most commonly affected site (58%). Laminotomy (72%) was the preferred surgical approach overall and specifically for exclusively intraspinal schwannomas (*p* = 0.02). GTR was achieved in 76.0% (*n* = 38). In multivariate analysis, only tumour localisation within the spinal canal (*p* = 0.014) independently predicted GTR, whereas the type of approach (*p* = 0.50) and tumour volume (*p* = 0.072) did not. New postoperative persisting deficits could not be predicted by any factor, including the use and alteration of intraoperative neuromonitoring. Recurrence was observed in four cases (8%) and was significantly higher in cases with STR (*p* = 0.04). *Conclusions:* In this retrospective study, GTR was solely predicted by tumour localisation within the spinal canal. The decision regarding the utilisation of different surgical approaches was solely influenced by the same factor. No factor could predict new persisting deficits. Tumour recurrence was higher in STR.

## 1. Introduction

Primary intraspinal tumours are a rare entity with an annual incidence of 1.5 per 100,000 persons. Of these, schwannomas are among the most common with an annual incidence of 0.3–0.4 per 100,000 habitants [1]. Spinal schwannomas are benign, slow-growing and encapsulated tumours that arise from Schwann cells [2,3]. The vast majority of spinal schwannomas are sporadic and solitary lesions; only 1% of schwannomas are related to neurofibromatosis type II (NF2), an autosomal dominant disorder characterised by the growth of benign tumours in the nervous system [4].

Spinal schwannomas are generally intra- or extradurally located and can present with various symptoms such as pain, radiculopathy and neurological deficit [5]. Schwannomas tend to arise from the sensory nerve roots and show a much rarer involvement of motor nerves, which could explain radiculopathy as a common presenting symptom [6]. The treatment of spinal schwannomas aims for complete resection with the preservation of neurovascular structures [5]. Complete resection may depend on various factors such as size, localisation and surgical accessibility.

Few studies have examined the factors that facilitate gross total resection (GTR) and to what extent subtotal resection (STR) leads to tumour recurrence in patients with non-syndromic spinal schwannomas [7]. In this context, the influence of the surgical approach has also been discussed [8]. Recent studies have evaluated the impact of the utilisation of intraoperative neuromonitoring (IONM) on the occurrence of new postoperative deficits, however, with contradictory results [9,10]. The aim of this single centre study was to identify factors affecting the resection rate of non-syndromic spinal schwannomas, as well as predictors of postoperative new deficits and factors affecting the surgeon’s decision regarding the surgical approach.

## 2. Materials and Methods

### 2.1. Study Population

A retrospective analysis of our dataset “Spinal neoplasms” was performed between January 2009 and December 2018. All the patients treated at our Neurosurgical Department due to a spinal schwannoma were included. Paediatric cases as well as patients with NF2 or schwannomatosis were excluded. The study has been carried out in accordance with The Code of Ethics of the World Medical Association (The Declaration of Helsinki) and was approved by the Institutional Review Board (Medical Faculty, University of Duisburg-Essen, Registration number: 20-9650-BO).

### 2.2. Data Management

Digital patients’ records were reviewed and the demographic parameters, presenting symptoms, surgical approach, necessity of instrumented stabilisation, neurological outcome and complications were noted for each case. In cases where intraoperative monitoring (IONM) was used, any decline of motor evoked potentials (MEP) and somatosensory evoked potentials (SSEP) was noted by neuromonitoring technicians. Postoperative new neurological deficits were defined as the occurrence of new neurological symptoms that were not present at preoperative examination and persisted at first follow-up. 

Pre- and postsurgical magnetic resonance (MR) imaging was examined, and tumour localisation, size and tumour volume were calculated using Gadolinium-enhanced T1-weighted images. For patients with clinical myelopathy, a concurrent spinal cord hyperintensity on T2-weighted MR images was determined. 

Multiple classifications for spinal schwannomas exist in the literature, each aiming to provide a certain surgical guidance based upon their categorisation [11,12,13]. Of these, Sun et al. have attempted to categorise spinal schwannomas according to their localisation as well as their size, and compared the surgical outcome for each category [14]. The authors provide a simple formula to estimate the size of schwannomas by considering them as ellipsoid bodies: tumour volume = 4/3π × (craniocaudal length/2) × transverse diameter/2)^2^.

**Figure 1:**
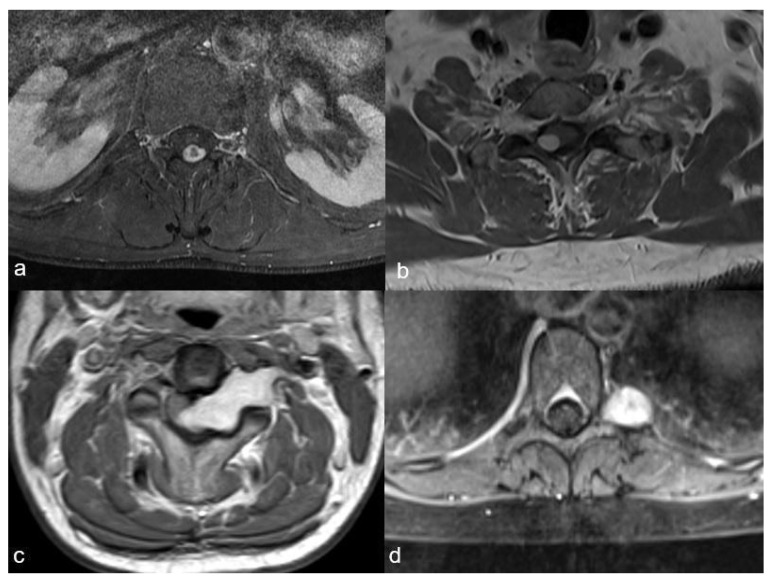
The four schwannoma localisations as described by Sun et al.: (**a**): exclusively intradural localisation (I), (**b**): intradural localisation with extradural extension to the nerve root foramina, but restricted to the spinal canal (II), (**c**): intradural tumour extending to the extraforaminal region (III), (**d**): localisation completely outside of the foramina (IV).

As this classification categorises schwannomas according to their localisation and size, we chose to apply it for our study. Spinal schwannomas were categorised accordingly into four distinct localisations (I, II, III and IV) and three sizes (A, B and C). Localisation I tumours were localised exclusively intradurally; Localisation II tumours had an intradural localisation with an extradural extension to the nerve root foramina but were restricted to the spinal canal; Localisation III tumours had an intradural dumbbell-shape in the spinal canal, extending to the extraforaminal region; and Localisation IV tumours were localised completely outside of the root foramina (Figure 1).

The patients were routinely followed-up every 6 months for 2 years after surgery. The follow-up included neurosurgical examination and spinal MR imaging with and without contrast agent. Tumour recurrence was defined as a new contrast-enhancing lesion at the site of the previous excision, identified on postoperative T1-weighted MR images.

### 2.3. Surgery

All the surgeries were performed with the use of a high-power magnification surgical microscope in the microsurgical fashion. The approach was chosen by the surgeon based on the localisation, size and type of the tumour. In cases where an en bloc laminoplasty was performed, the laminae were fixed in place using osteosynthesis material with mini fragment plates and screws (Promedics GmbH, Düsseldorf, Germany). Total resection of the schwannoma with the preservation of the nerve root and the surrounding tissues was attempted in all cases. En bloc resection was defined as isolating the tumour without violating its capsule, whereas intralesional resection implicated the violation of the tumor capsule, i.e., piecemeal resection [7]. The extent of the surgery was defined according to the surgeon’s assessment: GTR was defined as complete resection of the tumour from its attached nerve. Watertight dural closure was pursued in intradural tumours. Intraoperative neurophysiological monitoring (IONM) using MEP and SSEP was used in selected cases upon the individual surgeons’ decisions. The latency and amplitude of both median and tibial nerves were measured, and the surgeon was notified of any changes.

### 2.4. Endpoints

The primary endpoint was defined as GTR, whereas the secondary endpoints were defined as the surgical approach, new neurological deficit and tumour recurrence. Peri-procedural and mid-term complications were reported.

### 2.5. Statistics

Statistical analysis was performed with SPSS statistical software, version 23.0 (SPSS, Chicago, IL, USA). Factors influencing the primary and secondary endpoints of this study were first tested in univariate analysis. Continuous variables were tested with the Student’s *t*-test for normally distributed data and with the Mann–Whitney U test for non-normally distributed data. A chi-square test was used for dichotomised variables, and for samples with a size smaller than 5, Fisher’s exact test was used. Finally, a multivariate logistic regression analysis was performed for the primary endpoint to identify independent predictors upon identification in the univariate analysis and was corrected for the surgical approach. A *p*-value of <0.05 was considered statistically significant.

## 3. Results

### 3.1. Cohort Characteristics

Fifty patients met the inclusion criteria. The majority of the patients were males (64%), and the average age at presentation was 47 (±14) years. Radiculopathy was the most common symptom (88%). The interval between the first symptoms and surgical treatment was 16 (±35) months. The most common tumour localisation was lumbosacral (58%). Most schwannomas were smaller than 2 cm^3^. Schwannomas extending to the extraforaminal region (Localisation III schwannomas) were the most common type (36%). The average tumour volume was 9.7 (0.10–93.50) cm^3^, with 0.85 cm^3^ for Size A, 2.77 cm^3^ for Size B and 24.34 cm^3^ for Size C. Clinical myelopathy with concurrent spinal cord T2-hyperintensity on MR imaging was present in six (12%) cases. IONM was used in 22 cases (44%). Gross total resection, including *en-bloc* resections in 18 cases, was performed in the majority of the patients (76%), whereas STR was achieved in 12 cases (24%). Tumour recurrence occurred in one patient with GTR (2.6%) and three patients with STR (30%) (*p* = 0.04). The average follow-up was 30 (±27) months; five patients were lost to follow-up (Table 1).

### 3.2. Surgical Approach and Complications

The most common surgical approach was laminotomy (72%) including laminoplasty (54%) and laminectomy (18%). This technique provides a wide view on the posterior aspect of the spinal canal. A hemilaminectomy as a unilateral approach sufficed in 12 cases (24%). Instrumented stabilisation was performed in one case with a large C3 schwannoma, which was accessed via laminectomy of C3 and C4 and right-sided facetectomy. One case of a type IV mainly extraforaminal schwannoma at Th1 was operated via an extraforaminal approach. Surgical related complications occurred in five cases. Cerebrospinal fluid leakage was observed in four patients and required surgery in three cases. An epidural abscess occurred in one patient requiring the removal of the laminoplasty block.

### 3.3. Predictors of GTR

No significant differences were found in tumour localisation and resection rate. Univariate analysis showed that GTR was more common in Size A tumours (*p* = 0.004, odds ratio (OR) = 13.59, 95% confidence interval (95% CI) 1.59–116.3) and in Localisation I (*p* = 0.004, OR = 1.81, 95% CI 1.36–2.41), as well as in Localisation II (*p* = 0.05, OR = 1.41, CI 1.15–1.72) tumours. Respectively, STR was more common in Size C tumours (*p* < 0.001, OR = 0.04, CI 0.01–0.22) and Localisation III tumours (*p* < 0.001, OR 0.21, CI 0.02–0.19). Multivariate analysis identified Localisation III tumours (*p* = 0.014, adjusted (a) OR = 0.41, 95% CI 0.01–0.052) as an independent predictor for the extent of resection, but not the type of approach (*p* =0.50, aOR = 0.49, 95% CI 0.06–3.83) or Size C tumours (*p* = 0.072, aOR = 0.15, 95% CI 0.02–1.18).

### 3.4. Factors Influencing the Surgical Approach

In univariate analysis, the only factor impacting the chosen approach was for Localisation I tumours (72% laminotomy vs. 24% hemilaminectomy; *p* = 0.018, OR = 10.40, 95% CI 1.23–88.18). All other factors including tumour volume, location and myelopathy did not significantly affect the surgeon’s decision on the way of accessing the tumour (Table 2).

### 3.5. Predictors of New Postoperative Deficits

New persisting neurological deficits were noted in six cases: hypaesthesia in four cases, low grade paresis (Medical Research Council Scale 4) in two cases and neurological bladder, bowel and sexual dysfunction in one case with a giant lumbosacral schwannoma, complicated with postoperative cerebrospinal fluid leakage. Alterations during IONM occurred in three patients (MEP decline of the left tibial nerve in two patients and SSEP decline of the right median nerve in one patient) of which only one patient had a persisting new weakness in dorsiflexion as well as plantarflexion MRC 4. No alterations in MEP as well as SSEP were recorded in three patients that had a new persisting sensory deficit.

In univariate analysis, we were not able to identify any predictors of new persisting deficits among tumour location, size and type (Table 1). Furthermore, the approach as well as the use and alteration of IONM (*p* = 0.718, OR = 1.13, 95% CI 0.31–4.22 and *p* > 0.99, OR = 0.53, 95% CI 0.04–7.49, respectively) did not predict new persisting deficits.

## 4. Illustrative Case

**Figure 2:**
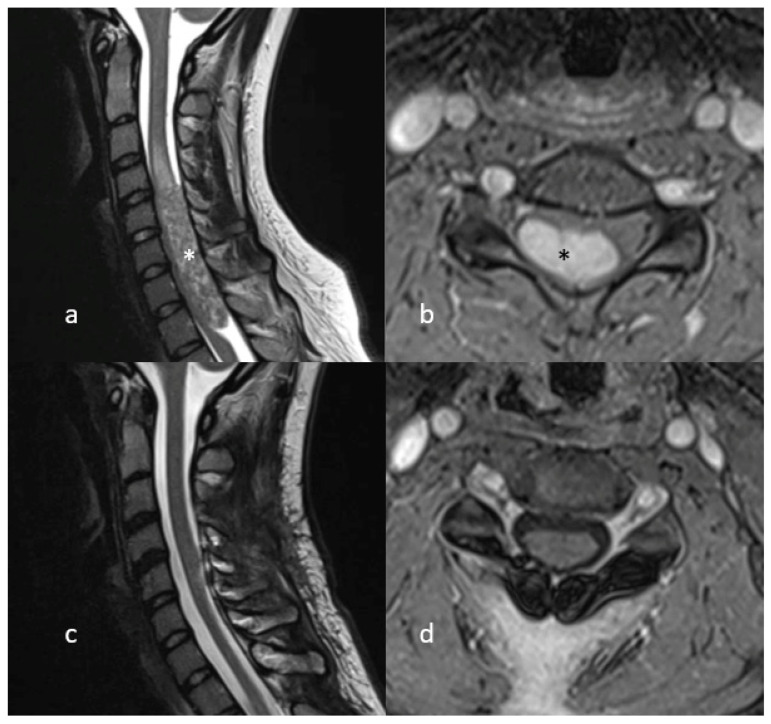
MR Imaging (T2-weighted on the left, T1 with gadolinium on the right) showing a large intradural, contrast-enhancing lesion (marked with asterisk) compressing the cervical spinal cord (**a**,**b**). Postoperative MR Imaging showing no signs of tumour recurrence (**c**,**d**).

**Figure 3:**
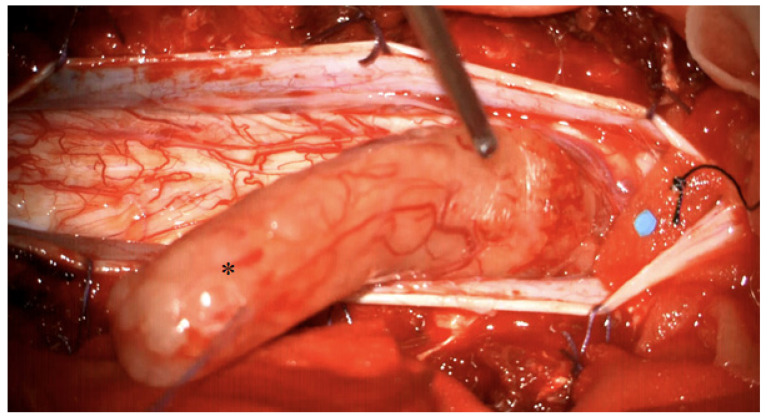
Intraoperative view after showing a large intradural schwannoma (marked with asterisk). The schwannoma is mobilised cranially, the compressed spinal cord is visible caudally.

A 24 year old female patient presented in our clinic complaining of neck pain and involuntary movements of both arms. No focal deficits were observed in the neurological examination. MR imaging showed a large intradural contrast-enhancing tumour, expanding from 5th cervical vertebra to the 1st thoracic vertebra, with intramedullary cord hyperintensity at T2-weighted MR imaging. The patient was positioned in a prone position and the head was fixed with the use of a Mayfield clamp. A laminotomy from the 4th cervical vertebra up to the 1st thoracic vertebra was performed. Midline durotomy was performed and the tumour was removed in toto with the help of an ultrasonic aspirator. A temporary decrease in SEP signals on both sides of up to 50% was noticed, whereas MEP signals remained constant during the whole procedure. After watertight dura closure, the laminotomy block was put into place and fixed with mini plates and screws. Postoperatively, the patient had no new focal neurological deficits. Eleven months of follow-up showed no tumour recurrence. Figure 2 and Figure 3.

## 5. Discussion

The aim of this study was to clarify which factors affect the resection rate of non-syndromic spinal schwannomas. We additionally sought to identify predictors for postoperative new deficits as well as factors affecting the surgeon’s decision regarding the surgical approach.

In this single centre retrospective cohort study of 50 patients, we identified that GTR was more likely in solely intraspinal located schwannomas and schwannomas smaller than 2 cm^3^. Consequently, STR was more common in schwannomas larger than >6 cm^3^ and in intraspinal schwannomas extending into the extraforaminal region. The same finding was observed in a large multicentre database, showing that tumour recurrence is higher in large sized tumours, with the extent in the cranial–caudal direction posing the greatest hazard [7]. However, it should be noted that GTR in larger tumours and tumours extending into the extraforaminal region may be possible with a more extensive surgery, requiring a second surgery and stabilisation.

Tumour localisation within the different spinal sections was not found to affect the resection rate. In our cohort, GTR was achieved in 76% of the cases, which included 18 cases with *en-bloc* resection of the tumour. This is within the range of previous reports [5,15,16,17]. In a previous study, *en-bloc* resection of spinal schwannomas in 34 patients resulted in new postoperative sensory deficits in 16 patients [5]. We observed no new deficits following *en-bloc* resection in our cohort study.

Tumour recurrence occurred in one patient in which GTR was performed, whereas three of the twelve patients that received a subtotal resection had tumour recurrence. Previous studies report a recurrence rate between 4% and 9% despite GTR [5,15,16,18]. No significant difference was found between en-bloc and intralesional resection in our study. In contrast, Fehlings et al. demonstrated a four-fold risk increase in tumour recurrence in patients who underwent intralesional resection [7]. It is noteworthy that GTR was according to the surgeon’s assessment which may yield a bias to the resection rate. Assessing the resection rate of benign spinal tumours via, e.g., early postoperative MR imaging has not been well described in the literature. Our study was performed in line with previous studies by assessing the resection rate via MR imaging after 3 months [7,16].

Laminotomy was identified as the preferred surgical approach in exclusively intradural schwannomas. This approach provides a broad view of the dorsal spinal cord and can be useful in the resection of tumours extending beyond the spinal canal. Hemilaminectomy, a technique reported as a safe and effective approach in spinal tumour resection, sufficed in 24% (12 cases) [19]. None of the other factors such as tumour volume, location and myelopathy significantly affect the surgeon’s decision on the way of accessing the tumour. It should be emphasised that surgical approach can be institution- and surgeon-dependent. The use of minimally invasive techniques and endoscopic approaches may be useful in selected cases [20,21].

New persisting neurological deficits were noted in six cases. Tumour location, size and type were not a predictor for persisting new deficits. Furthermore, the approach as well as the use and alteration of IONM did not predict new persisting deficits. IONM is recommended for resections of intra- and extramedullary tumours, however, the added value during excision of schwannomas has been debated [10,22,23]. Although some authors report an increase in safety during spinal schwannoma surgery, some authors state that intraoperative monitoring is not able to detect roots vulnerable to injury [9]. We observed the same finding, as none of the patients with intraoperative signal decline had permanent neurological deficits and as one patient without any signal decline had a new persisting sensory deficit. The added value of IONM in schwannoma surgery requires further research.

This cohort study has several limitations. First, the retrospective art of the study may have resulted in selection and information bias. Second, the method used for tumour size calculation assumes that all spinal schwannomas are ellipsoid-shaped. Third, the recurrence rate may also depend on proliferation indices such as Ki-67, which was not investigated in our study as this factor was not determined for each case [15]. Fourth, the average follow-up was 30 months and five patients were lost to follow-up. Because of the slow-growing attribute of spinal schwannomas, more extensive follow-up is advisable.

## 6. Conclusions

Our findings suggest that GTR is more likely to be achieved in smaller schwannomas independent of localisation. The surgical approach did not affect the resection rate. STR is observed in tumours extending into the neuroforamen and is associated with a higher tumour recurrence rate. New postoperative persisting deficits could not be predicted by any factor, including the use and alteration of IONM. Future prospective clinical studies are required for a more accurate understanding of the factors contributing to GTR in spinal schwannomas as well as recurrence rates in STR. Finally, IONM was not used in each individual case.

## Figures and Tables

**Table 1:** Patient and tumour characteristics.

		*n*	%
Gender	Female	18	36
Age (years) ^a^		46 (±14)	
Clinical presentation	Symptoms related to tumor:	46	92
	Radiculopathy ^b^	44	88
	Spinal deficit	11	22
	Unrelated symptom(s) leading to diagnosis	4	8
Time between first symptoms and diagnosis (months)		16 (35)	
Tumour characteristics (MR imaging)			
Tumour localisation	Cervical	15	30
	Thoracal	6	12
	Lumbosacral	29	58
	Central	9	18
	Lateral:	41	82
	Right	21	42
	Left	20	40
Volume of tumour (cm^3^)	Mean volume ^a^	9.7 (18.8)	
	Size A (0–2)	22	44
	Size B (2–6)	12	24
	Size C (>6)	16	32
Localisation type	I	17	34
	II	11	22
	III	18	36
	IV	4	8
Presence of myelopathy	yes	6	12

^a^ Mean (SD); ^b^ Radiculopathy was defined as paresis, hypaesthesia, paraesthesia as well as dysaesthesia.

**Table 2:** Univariate analysis.

*Primary Endpoints*					
	*GTR*	*STR*	*p-Value*	*OR*	*95% CI*
Tumour size					
**A**	**21**	**1**	**0.004**	**13.59**	**1.59–116.03**
B	11	1	0.248	4.48	0.52–39.01
**C**	**6**	**10**	**<0.001**	**0.04**	**0.01–0.22**
Localisation type					
**I**	**17**	**0**	**0.004**	**1.81**	**1.36–2.41**
**II**	**11**	**0**	**0.046**	**1.41**	**1.15–1.72**
**III**	**7**	**11**	**>0.001**	**0.21**	**0.02–0.19**
IV	3	1	>0.99	0.94	0.09–10.01
*Secondary endpoints*					
	*Laminotomy*	*Hemilaminectomy*			
Tumour size					
A	16	6	0.919	1.07	0.31––3.71
B	9	3	>0.99	1.22	0.28–5.38
C	11	5	0.746	0.79	0.22–2.92
Localisation type					
**I**	**16**	**1**	**0.018**	**10.40**	***1.23–88.18***
II	9	2	0.705	2.00	0.37–10.69
III	10	8	0.052	0.29	0.08–1.04
IV	1	3	0.061	0.11	0.01–1.11
Myelopathy	5	1	0.663	2.01	0.22–19.75
	*Persisting deficits* *(n = 6)*	*No new deficits* *(n = 44)*			
IONM	2	20	0.683	0.60	0.10–3.62
IONM alteration	0	3	>0.99	1.00	0.04–25.69
Resection intralesional	4	29	>0.99	1.03	0.17–6.31
Myelopathy	0	6	>0.99	0.52	0.02–9.10
GTR	4	34	0.621	0.588	0.09–3.67
Tumour size					
A	2	20	0.683	0.60	0.10–3.62
B	2	10	0.621	1.70	0.27–10.68
C	2	14	>0.99	1.07	0.18–6.56
Tumour localisation					
I	2	9	0.601	1.94	0.31–12.35
II	3	15	0.654	1.93	0.35–10.77
III	0	4	>0.99	0.69	0.03–14.43
IV	2	9	0.601	1.94	0.31–12.35

## Data Availability

The authors confirm that the raw data that support the findings of this study are available from the corresponding author upon request.

## Abbreviations

Gross total resection	GTR
Magnetic resonance imaging	MRI
Neurofibromatosis type II	NF2
Subtotal resection	STR
Intraoperative neuromonitoring	IONM
MEP	motor evoked potentials
SSEP	somatosensory evoked potentials
